# Combined sedation in pediatric magnetic resonance imaging: determination of median effective dose of intranasal dexmedetomidine combined with oral midazolam

**DOI:** 10.1186/s12871-024-02493-x

**Published:** 2024-03-23

**Authors:** Hao Xie, Jialian Zhao, Haiya Tu, Wenyang Wang, Yaoqin Hu

**Affiliations:** Department of Anesthesiology, Children’s Hospital, School of Medicine, Zhejiang University, 3333 Binsheng Rd, Hangzhou, Zhejiang 310052 P.R. China

**Keywords:** Combined sedation, Intranasal dexmedetomidine, Oral midazolam, Pediatric magnetic resonance imaging, Median effective dose

## Abstract

**Background:**

The exact median effective dose (ED50) of intranasal dexmedetomidine combined with oral midazolam sedation for magnetic resonance imaging (MRI) examination in children remains unknow and the aim of this study was to determine the ED50 of their combination.

**Methods:**

This is a prospective dose-finding study. A total of 53 children aged from 2 months to 6 years scheduled for MRI examination from February 2023 to April 2023 were randomly divided into group D (to determine the ED50 of intranasal dexmedetomidine) and group M (to determine the ED50 of oral midazolam). The dosage of dexmedetomidine and midazolam was adjusted according to the modified Dixon’s up-and-down method, and the ED50 was calculated with a probit regression approach.

**Results:**

The ED50 of intranasal dexmedetomidine when combined with 0.5 mg∙kg^− 1^ oral midazolam was 0.39 µg∙kg^− 1^ [95% confidence interval (CI) 0.30 to 0.46 µg∙kg^− 1^] while the ED50 of oral midazolam was 0.17 mg∙kg^− 1^ (95% CI 0.01 to 0.29 mg∙kg^− 1^) when combined with 1 µg∙kg^− 1^ intranasal dexmedetomidine. The sedation onset time of children with successful sedation in group D was longer than in group M (30.0[25.0, 38.0]vs 19.5[15.0, 35.0] min, *P* < 0.05). No other adverse effects were observed in the day and 24 h after medication except one dysphoria.

**Conclusion:**

This drug combination sedation regimen appears suitable for children scheduled for MRI examinations, offering a more precise approach to guide the clinical use of sedative drugs in children.

**Trial registration:**

Chinese Clinical Trial Registry, identifier: ChiCTR2300068611(24/02/2023).

**Supplementary Information:**

The online version contains supplementary material available at 10.1186/s12871-024-02493-x.

## Introduction

Sedation is frequently necessary for diagnostic procedures in children who struggle to cooperate, such as magnetic resonance imaging (MRI), computed tomography (CT), auditory brainstem response (ABR), and transthoracic echocardiography (TTE). The effective dosage likely differs among different procedures based on their duration, invasiveness, and need for varying levels of depth of sedation. Performing MRI in children under 6 years old is often challenging due to high noise, long examination times, the required immobility, and the need for sedatives, resulting in a certain failure rate [1–3].

Intranasal dexmedetomidine is widely employed for pediatric sedation due to its safety, non-invasiveness, and convenience. It produces sedative effects akin to natural sleep with fewer respiratory depression events [4–9]. However, using dexmedetomidine alone may necessitate higher doses, prolong sedation times, reduce success rates, and increase side effects [10, 11]. Consequently, a combination regimen of intranasal dexmedetomidine and oral midazolam, which is a type of short-acting benzodiazepine with anxiolytic, hypnotic, anterograde amnestic effects [12, 13], is increasingly utilized for its advantages, including higher success rates and fewer complications [14–16].

Nevertheless, the effective dosage of intranasal dexmedetomidine combined with oral midazolam for sedation during MRI examinations in children has not been reported. This study aims to determine the median effective dose (ED50) of intranasal dexmedetomidine combined with oral midazolam sedation for children before MRI examinations using a modified Dixon’s up-and-down method [17].

## Methods

### Study design and ethical approval

The study adhered to the Declaration of Helsinki (2013) and was approved by the medical ethics committee of Children’s Hospital, Zhejiang University, School of Medicine (reference number 2022-IRB-275, Chairperson Professor Gong Fangqi, 26 December 2022). It was registered in the Chinese Clinical Trail Registry (ChiCTR 2,300,068,611) before subject enrollment. Written informed consent was obtained from all legal guardians. In this single-center dose-finding experimental study, children were randomly assigned by random number table to Group D (to determine the ED50 of intranasal dexmedetomidine combined with a fixed dose of oral midazolam) or Group M (to determine the ED50 of oral midazolam combined with a fixed dose of intranasal dexmedetomidine).

### Patient enrollment

Pediatric patients aged 2 months to 6 years, scheduled for sedation before MRI with an American Society of Anesthesiology (ASA) score of I–II, were eligible for enrollment. Exclusion criteria included lack of consent, allergy to dexmedetomidine or midazolam, severe arrhythmias (including high-grade atrioventricular block, supraventricular tachycardia and frequent ventricular premature beat, etc.), nasal mucosal injury, severe upper respiratory tract infection (accompanied by a fever(≥38.5℃), an intense cough and sputum, purulent nasal discharge, shortness of breath, lung rales or other symptoms), mental awareness disorder, hepatic or renal dysfunction, obesity (age 2–6 years: body mass index(BMI) above the obesity reference threshold on the BMI growth curve of children; < 2 years old: 3 standard deviations greater than the mean weight of the reference population), and follow-up missing.

### Modified Dixon’s up-and-down method

Based on existing literature [18–20] and our pretest study, we used the modified Dixon’s up-and-down method to determine the median effective dose of dexmedetomidine and midazolam when used in combination [21]. Patients were randomly assigned to the Group D or Group M.

Group D: Oral midazolam was administered at a constant dose of 0.5 mg kg^− 1^, and the initial dose of intranasal dexmedetomidine was 0.5 µg kg^− 1^. The dexmedetomidine dose was adjusted by 0.1 µg kg^− 1^ based on sedation success or failure in the previous patient.

Group M: Intranasal dexmedetomidine was given at a constant dose of 1 µg kg^− 1^, and the initial dose of oral midazolam was 0.25 mg kg^− 1^. The midazolam dose was adjusted by 0.05 mg kg^− 1^ based on sedation success or failure in the previous patient.

Children in each group were recruited until eight crossovers (from failed sedation to successful sedation) were achieved, and at least 20 children were included [21].

### Sedation procedure

#### Before sedation

All children followed the same fasting guidelines [22]: fasting from clear liquids for 2 h, breast milk for 4 h, light meal or infant formula for 6 h, and fried or fatty foods or meat for 8 h. Routine necessary medications were allowed with a sip of clear liquid on the day of the MRI examination. There was no requirement for the awake time of children before sedation. Baseline vital signs, including blood pressure (BP), heart rate (HR), and oxyhemoglobin saturation (SpO2), were measured before drug administration.

#### Sedation administration and definitions

Fifty minutes before the MRI examination, children received intranasal dexmedetomidine (Jiangsu Hengru Medicine CO., Ltd., China, batch No. 220527BP, 2 ml: 200 ug) followed by oral midazolam (Yichang Humanwell Pharmaceuticals, China, batch No. 1L911011,10 ml: 20 mg), administered by a sedation nurse unaware of the group and drug dosage. Sedation was assessed using the Modified Observer Assessment of Alertness and Sedation (MOAA/S) scale [23] every 10 min before the eyes were closed and every 1–2 min after the eyes were closed. Successful sedation was defined as MOAA/S ≤ 2 five minutes before the MRI examination and during the examination, while failed sedation was defined as MOAA/S > 2 or the child waking up during the examination. Inhalation sevoflurane or intravenous propofol was administered for rescue sedation.

Overall sedation time was defined as the time from drug administration to reaching the Modified Aldrete Score (MAS) [14] ≥ 8. Sedation onset time was from drug administration to the beginning of MOAA/S ≤ 2. Sedation recovery time was defined as the time interval from achieving MOAA/S ≤ 2 to awakening without disturbance, with delayed awakening defined as recovery time greater than 120 min [1].

#### Sedation recovery and follow-up

After completing the MRI examination, the child was observed in the recovery room. Recovery assessment based on MAS was conducted by another uninformed anesthesiologist. When MAS was ≥ 8, the child could drink clear water and eat normally, and they could leave the hospital with MAS ≥ 9 and no adverse reactions after eating for 20 min. Children and guardians were followed up by telephone the next day to record adverse events such as nausea, vomiting, dysphoria, and drowsiness.

#### Monitoring

Data recorded included demographic information, vital signs, sedation scores, sedation onset time, sedation recovery time, overall sedation time, and occurrence of complications. All data collection was performed by an anesthetist unaware of the drug administration method.

### Statistical analysis

The median effective dose was determined by Dixon’s up-and-down method, which calculates the mean of the crossover midpoints. Probit regression analysis enabled us to calculated ED50 and ED95 and estimated 95% confidence intervals(95%CI) [24].

Statistical analysis utilized SPSS 22.0 for Windows (SPSS Inc., Chicago, IL, USA). Normal distributed data were presented as mean [standard deviation (SD)], and non-normally distributed data were expressed as median [interquartile range (IQR)]. Statistical analysis for differences between the groups was compared by the two-tailed Student’s t-test when normality (and homogeneity of variance) assumptions were satisfied, otherwise the nonparametric test (Mann–Whitney U) was used. Categorical data were analyzed by chi-square test or Fisher’s exact test. The threshold for statistical significance was set at *P* < 0.05.

## Results

**Fig. 1:**
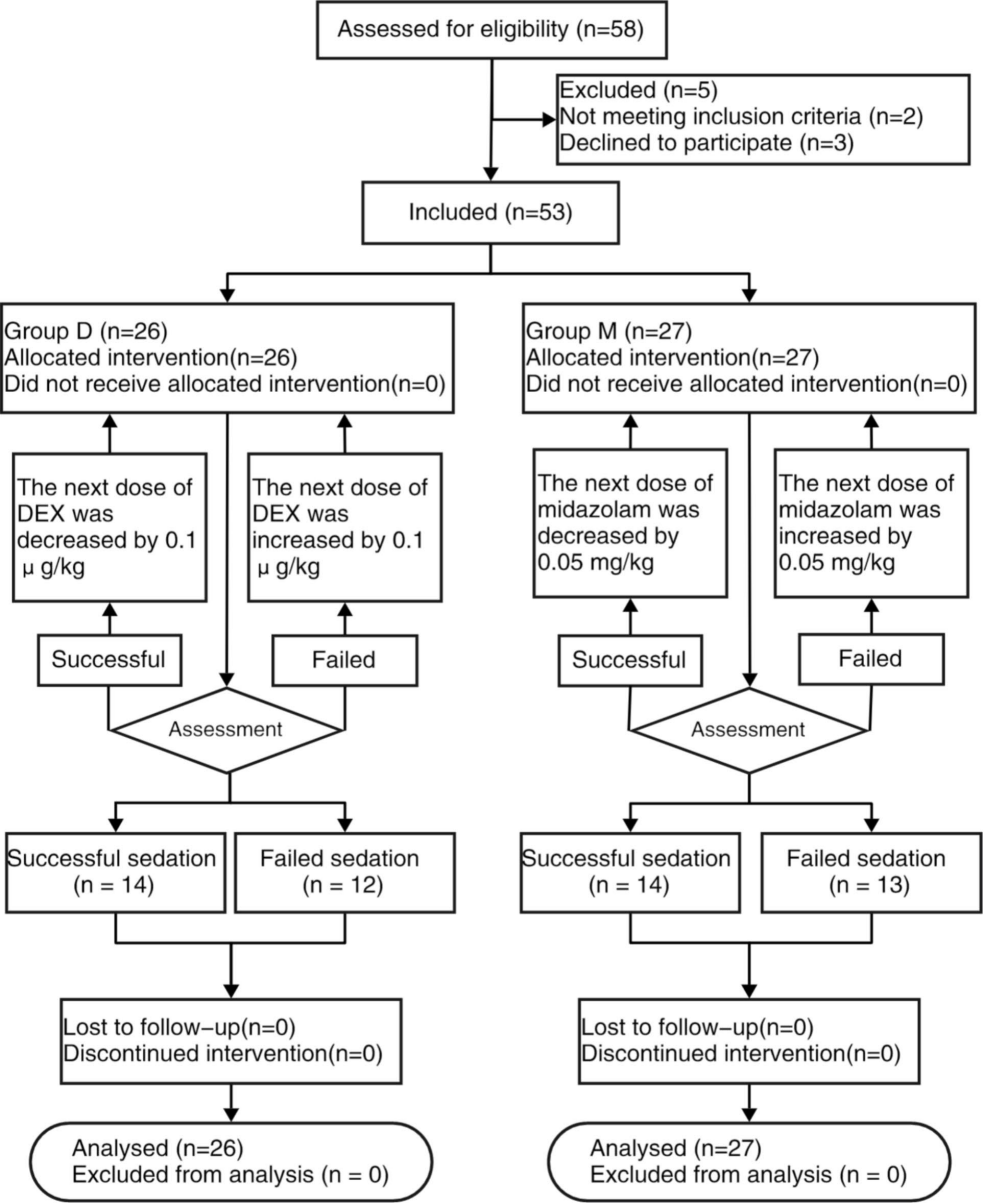
CONSORT flow diagram of the study. Group D was designed to determine the ED50 of intranasal dexmedetomidine. Group M was designed to determine the ED50 of oral midazolam. CONSORT, Consolidated Standards of Reporting Trials. DEX, dexmedetomidine

A total of 58 children scheduled for MRI examination from 28 February 2023 to 28 April 2023 were assessed for eligibility, and 53 patients were enrolled in this prospective study (Fig. 1). The demographic data (sex, age, weight, and ASA classification) were listed in Table 1 showing no significant difference between the two groups (*p* > 0.05). The sedation onset time of children with successful sedation in group D was longer than in group M(*p* < 0.05) but there was no significant difference in examination time, sedation recovery time and overall sedation time between the two groups (*P* > 0.05) (Table 1).

**Table 1:** Characteristics of children receiving intranasal dexmedetomidine combined with oral midazolam sedation for MRI examination

	Group D(n = 26)	Group M(n = 27)	*P*-value
Male/Female(n/n)	12/14	17/10	0.219
Age (month)	18 [6, 36]	37[8, 49]	0.109
Weight (kg)	12.06 ± 4.84	14.19 ± 5.69	0.149
ASA (I/II)	21/5	25/2	0.250
Successful sedation(n)	14	14	
Examination time (min)	7.0[7.0, 10.0]	7.0[6.8, 8.0]	0.217
Sedation onset time (min)	30.0[25.0, 38.0]	19.5[15.0, 35.0]	**0.027**
Sedation recovery time (min)	81.2 ± 26.0	73.6 ± 28.3	0.463
Overall sedation time (min)	112.4 ± 23.0	98.2 ± 30.0	0.171

*Notes* Data are presented as the number of patients (n/n), median [IQR] or mean ± SD

*Abbreviations* ASA, American Society of Anesthesiology; SD, standard deviation; IQR, interquartile range

**Fig. 2:**
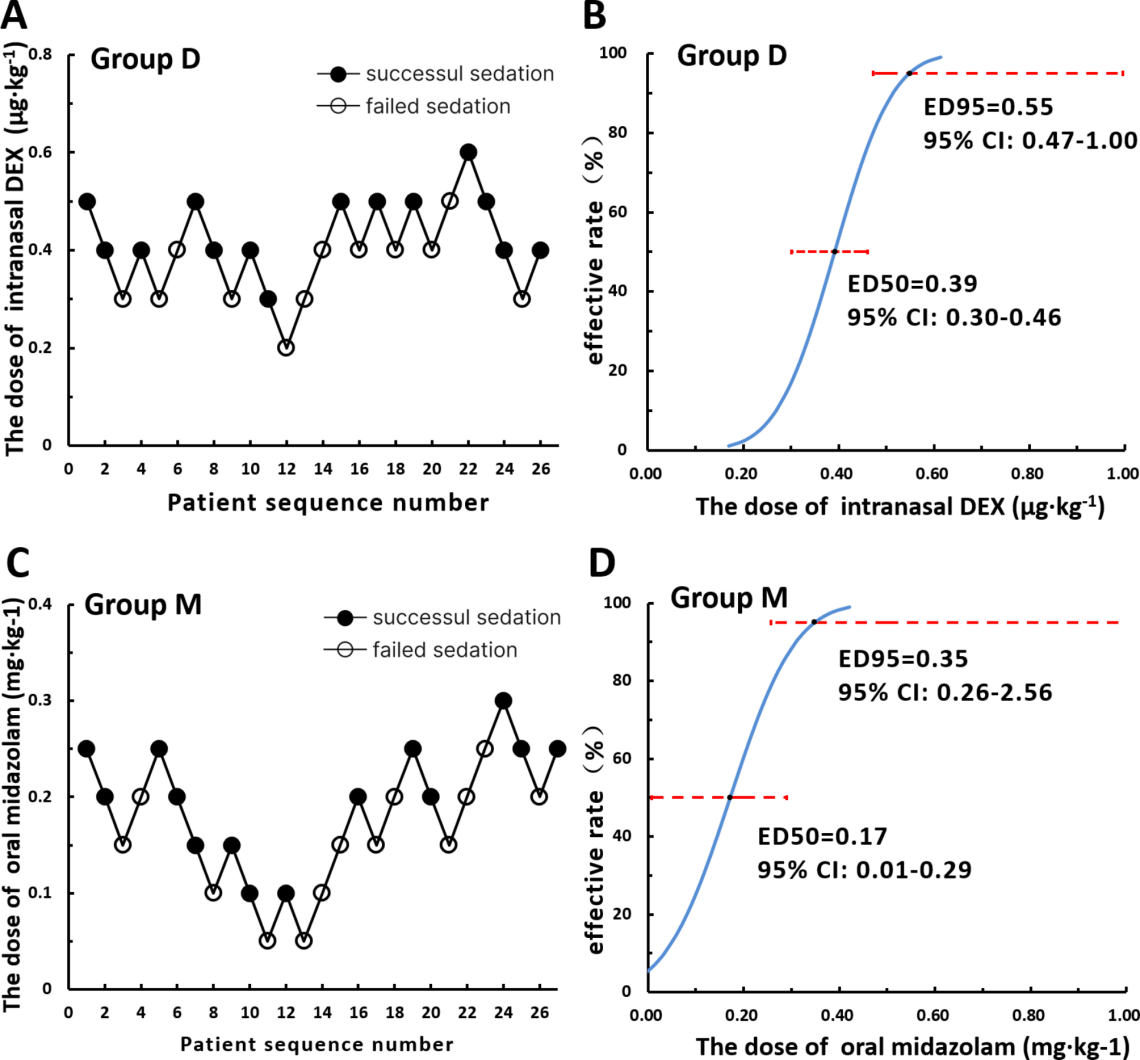
Sequential diagrams and dose-effect diagrams of intranasal dexmedetomidine and oral midazolam in sedation for pediatric MRI examination in group D and group M. In the sequential diagrams (**A** and **C**), the patient sequence number (X-axis) is the order of child exposures using the modified Dixon’s up-and-down method. The assigned dose levels are presented on the Y-axis. A successful sedation dose is denoted by a solid circle, while a failed sedation dose is denoted by a hollow circle. Dose-effect curves of intranasal dexmedetomidine and oral midazolam (**B** and **D**) indicate the effective rate of sedation (X-axis) related to the drug dose (Y-axis) by using probit analysis. The ED50 (95% CI) and ED95 (95% CI) of intranasal dexmedetomidine were 0.39 µg kg^− 1^ (95% CI 0.30 to 0.46 µg kg^− 1^) and 0.55 µg kg^− 1^ (95% CI 0.47 to 1.00 µg kg^− 1^), respectively, in group D. The ED50 (95% CI) and ED95 (95% CI) of oral midazolam for sedation were 0.17 mg kg^− 1^ (95% CI 0.01 to 0.29 mg kg^− 1^) and 0.35 mg kg^− 1^ (95% CI 0.26 to 2.56 mg kg^− 1^), respectively, in group M. Group D was designed to determine the ED50 of intranasal dexmedetomidine. Group M was designed to determine the ED50 of oral midazolam. DEX, dexmedetomidine; CI, confidence interval; ED50, median effective dose; ED95, 95% effective dose; MRI, magnetic resonance imaging

The sequence of children with failed sedation (hollow circle) and successful sedation (solid circle) and the dose-effect relationship obtained by probit regression analysis are depicted in Fig. 2. The ED50 (95% CI) and ED95 (95% CI) of intranasal dexmedetomidine for sedation were 0.39 µg kg^− 1^ (95% CI 0.30 to 0.46 µg kg^− 1^) and 0.55 µg kg^− 1^(95% CI 0.47 to 1.00 µg kg^− 1^) when combined with 0.5 mg kg^− 1^ dose of oral midazolam. Meanwhile, the ED50 (95% CI) and ED95 (95% CI) of oral midazolam for sedation were 0.17 mg kg^− 1^ (95% CI 0.01 to 0.29 mg kg^− 1^) and 0.35 mg kg^− 1^ (95% CI 0.26 to 2.56 mg kg^− 1^) when combined with 1 µg kg^− 1^ dose of intranasal dexmedetomidine. There were 12 (46.2%) failed sedations in group D and 13 (48.1%) failed sedations in group M, which were rescued with inhalation sevoflurane or intravenous propofol.

**Fig. 3:**
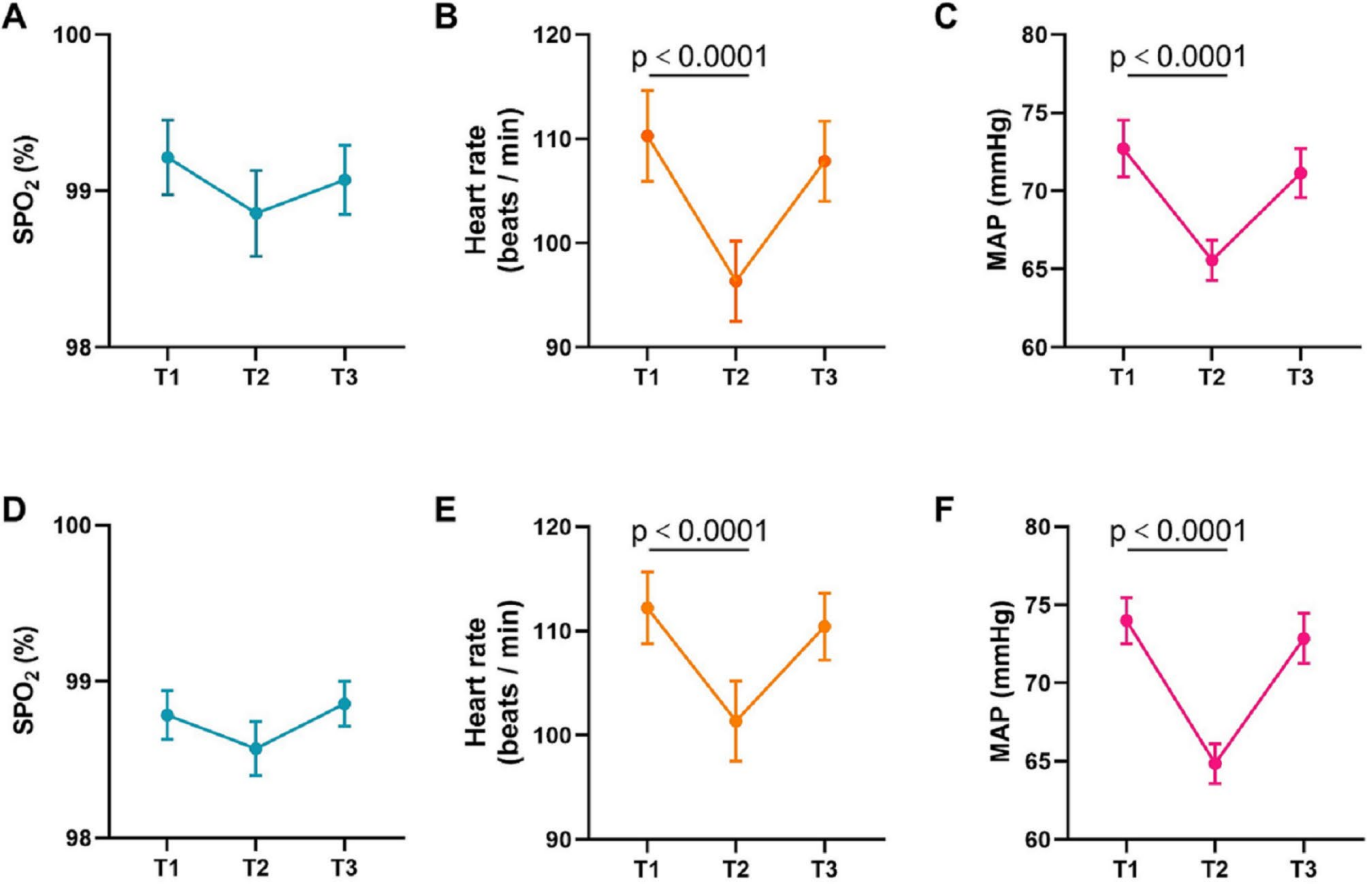
Hemodynamic changes in the children across different study time points in the two groups. (**A**, **B** and **C**), Changes in SPO2, HR, and MAP across different study time points in group D. (**D**, **E** and **F**), Changes in SPO2, HR, and MAP across different study time points in group M. T1, baseline; T2, at the time after sedation onset; T3, at the time after awakening. Group D was designed to determine the ED50 of intranasal dexmedetomidine. Group M was designed to determine the ED50 of oral midazolam. SpO2, oxyhemoglobin saturation; HR, heart rate; MAP, mean arterial pressure

The hemodynamics of the children are illustrated in Fig. 3. The HR and MAP of children in the two groups were both significantly lower after sedation onset than at baseline (*p* < 0.0001), and these values remained within the normal range. There were no measurable changes of the vital signs after awakening compared to premedication in the two groups (*p* > 0.05). Dysphoria was observed in a 3-month-old child during the recovery in group D, which subsided after breastfeeding. No other adverse effects were reported within 24 h after medication.

## Discussion

This prospective, single-center, dose-finding study investigated intranasal dexmedetomidine combined with oral midazolam sedation for children during MRI examinations in a large tertiary children’s hospital in China. The results revealed that the ED50 and ED95 of intranasal dexmedetomidine in Group D were 0.39 µg kg^− 1^ (95% CI: 0.30 to 0.46 µg kg^− 1^) and 0.55 µg kg^− 1^ (95% CI: 0.47 to 1.00 µg kg^− 1^), and the ED50 and ED95 of oral midazolam in Group M were 0.17 mg kg^− 1^ (95% CI: 0.01 to 0.29 mg kg^− 1^) and 0.35 mg kg^− 1^ (95% CI: 0.26 to 2.56 mg kg^− 1^). On this basis, the smallest recommended dose of intranasal dexmedetomidine was 0.55 µg kg^− 1^ when combined with 0.5 mg kg^− 1^ oral midazolam while the smallest recommended dose of oral midazolam was 0.35 mg kg^− 1^ when combined with 1 µg kg^− 1^ intranasal dexmedetomidine. Polypharmacy has been associated with increased risk of adverse events and the reduction of drug dose may lead to a decrease in the incidence of adverse reactions [25]. Compared to reported medication regimens, the recommended doses in this study were significantly lower than those used by Cozzi et al. [26], suggesting potential safety benefits with fewer adverse effects. These inferences still need to be tested by further research.

Dixon’s up-and-down method, commonly employed in anesthesia dose-finding research, was utilized to explore the median effective dose [17]. Görges et al. [27] confirmed the value of the such sequential allocation trial design and indicated that the main advantage was the potential for reduced sample sizes and, in particular, minimizing the exposure of subjects to suboptimal treatments. In our study, the ED50 and ED95 were determined by the Dixon’s up-and-down method and probit regression, allowing for the evaluation of drug efficacy with fewer cases over a shorter time [28]. The selected doses of oral midazolam in Group D and intranasal dexmedetomidine in Group M (0.5 mg kg^− 1^ and 1 µg kg^− 1^, respectively) were lower than those used as sole agents for children based on previous studies [18, 19].

In our study, the sedation onset time of children with successful sedation in Group D was significantly longer than that in Group M. There are several possible explanations. Van Groen et al. [29] reported a lower typical oral bioavailability of midazolam (66%) compared to the overall intranasal dexmedetomidine bioavailability of 84% [5] due to first-pass hepatic metabolism. Additionally, intranasal dexmedetomidine demonstrated a significantly higher incidence of satisfactory sedation compared to oral midazolam [30, 31]. The higher dose of intranasal dexmedetomidine in Group M (1 µg kg^− 1^) than in Group D (0.2–0.6 µg kg^− 1^) possibly resulted in this difference in sedation onset time.

Notably, our study showed a longer sedation recovery time compared to previous studies [32]. This difference was attributed to our approach of allowing children to wake up naturally without stimulation during the recovery stage, aiming to avoid discomfort after forced awakening.

Both dexmedetomidine and midazolam may reduce heart rate and blood pressure, with dexmedetomidine having a greater impact on heart rate than midazolam [33, 34]. However, our study, similar to previous research [14, 35–37], demonstrated no serious hemodynamically unstable changes requiring clinical intervention. It is well-known that higher midazolam and dexmedetomidine doses generally lead to a higher incidence of adverse events of sedation [10, 18]. No respiratory complications occurred in our study, likely attributed to the low drug doses. Bellolio et al. [38] identified vomiting as the most common adverse effect of sedation in children. To avoid regurgitation and vomiting, all enrolled children were asked to follow the same fasting guidelines as those for general anesthesia, which could disturb sedative effect and increase the incidence of dysphoria because of the sense of hunger and thirst, especially in young children. In our study, the child with dysphoria may be related to this.

Some limitations merit discussion. Our inclusion of children under 6 years old without age stratification may impact the sedative effect, considering potential age-related variations in drug response [39–41]. The MRI examination time in our study was short due to the single-site examination such as head magnetic resonance plain scan and the results of this study may not be applicable to long duration MRI examination. As a single-center study with a limited sample size, the wide 95% confidence intervals of the ED95 indicate uncertainty, necessitating further rigorous prospective studies with larger samples for validation.

## Conclusions

In our study, the ED50 of intranasal dexmedetomidine and oral midazolam for sedation in children scheduled for MRI examinations was estimated. This drug combination sedation regimen appears suitable for children scheduled for MRI examinations, offering a more precise approach to guide the clinical use of sedative drugs in children.

### Electronic supplementary material

Below is the link to the electronic supplementary material.

Supplementary Material 1

## Data Availability

The datasets used and analyzed during the current study are available from the corresponding author upon reasonable request.

## Abbreviations

ABR: auditory brainstem response
ASA: American Society of Anesthesiology
BMI: body mass index
CI: confidence interval
CT: computed tomography
ED50: median effective dose
ED95: the 95% effective dose
IQR: interquartile range
MAS: Modified Aldrete Score
MOAA/S: Modified Observer Assessment of Alertness and Sedation
MRI: magnetic resonance imaging
SD: standard deviation
TTE: transthoracic echocardiography
