# Supplementary material for: Combined sedation in pediatric magnetic resonance imaging: determination of median effective dose of intranasal dexmedetomidine combined with oral midazolam
Source: BMC Anesthesiol. 2024 Mar 23;24:112. doi: 10.1186/s12871-024-02493-x (PMC10960491; doi:10.1186/s12871-024-02493-x)
Supplement: Supplementary file 1 — Supplementary Material 1 [file 12871_2024_2493_MOESM1_ESM.docx]

**Table 1**. Appropriate Intake of Food and Liquids Before Sedation

| **Ingested Material** | **Minimum Fasting Period, h** |
| --- | --- |
| Clear liquids: water, fruit juices without pulp, carbonated beverages, clear tea, black coffee(no alcohol). | 2 |
| Breast milk | 4 |
| A light meal or nonhuman milk | 6 |
| Fried foods or fatty foods or meat | 8 |

**Table 2**. Modifified observer's assessment of alertness/sedation scale.(MOAA/S Scale)

| **MOAA/S Scale** | **Scale** |
| --- | --- |
| Responds readily to name spoken in normal tone | 5 |
| Lethargic response to name spoken in normal tone | 4 |
| Responds only after name is called loudly and/or repeatedly | 3 |
| Responds only after mild prodding or shaking | 2 |
| Responds only after painful trapezius squeeze | 1 |
| Does not respond to painful trapezius squeeze | 0 |

**Table 3** Modifed Aldrete score(MAS)

| **Response** | | **Score** |
| --- | --- | --- |
| **Consciousness** |  |  |
|  | Fully awake | 2 |
|  | Arousable | 1 |
|  | Not responding | 0 |
| **Breathing** |  |  |
|  | Able to breathe deeply | 2 |
|  | Dyspnea | 1 |
|  | Apnea | 0 |
| **Mobility** |  |  |
|  | Able to move four extremities on command | 2 |
|  | Able to move two extremities on command | 1 |
|  | Able to move zero extremities on command | 0 |
| SpO_2_ |  |  |
|  | Maintaining O2 saturation>92% on room air | 2 |
|  | Needing inhalation to maintain O2 saturation>92% | 1 |
|  | O2 saturation<92% despite O2 supplementation | 0 |
| **Circulation** |  |  |
|  | Systemic blood pressure<20% of the preanesthetic level | 2 |
|  | Systemic blood pressure between 20%–49% of the preanesthetic level | 1 |
|  | Systemic blood pressure ≥ 50% of the preanesthetic level | 0 |
